# Phase I/II trial of meclofenamate in progressive MGMT-methylated glioblastoma under temozolomide second-line therapy—the MecMeth/NOA-24 trial

**DOI:** 10.1186/s13063-021-05977-0

**Published:** 2022-01-19

**Authors:** Thomas Zeyen, Anna-Laura Potthoff, Robert Nemeth, Dieter H. Heiland, Michael C. Burger, Joachim P. Steinbach, Peter Hau, Ghazaleh Tabatabai, Martin Glas, Uwe Schlegel, Oliver Grauer, Dietmar Krex, Oliver Schnell, Roland Goldbrunner, Michael Sabel, Niklas Thon, Daniel Delev, Hans Clusmann, Clemens Seidel, Erdem Güresir, Matthias Schmid, Patrick Schuss, Frank A. Giordano, Alexander Radbruch, Albert Becker, Johannes Weller, Christina Schaub, Hartmut Vatter, Judith Schilling, Frank Winkler, Ulrich Herrlinger, Matthias Schneider

**Affiliations:** 1 Division of Clinical Neurooncology, Department of Neurology and Center of Integrated Oncology, University Hospital Bonn, Bonn, Germany; 2 Department of Neurosurgery and Center of Integrated Oncology, University Hospital Bonn, Bonn, Germany; 3 Institute of Medical Biometry, Informatics and Epidemiology, University Hospital Bonn, Bonn, Germany; 4 Department of Neurosurgery, University of Freiburg, Freiburg, Germany; 5 Faculty of Medicine, University of Freiburg, Freiburg, Germany; 6 Dr. Senckenberg Institute of Neurooncology, Goethe-University Hospital, Frankfurt am Main, Germany; 7 Department of Neurology and Wilhelm Sander-NeuroOncology Unit, University Hospital Regensburg, Regensburg, Germany; 8 Interdisciplinary Division of Neurooncology, University of Tübingen, Tübingen, Germany; 9 Division of Clinical Neurooncology, Department of Neurology, University Hospital Essen, Essen, Germany; 10 Department of Neurology, University Hospital Knappschaftskrankenhaus, Ruhr-Universität Bochum, Bochum, Germany; 11 Department of Neurology, University of Münster, Münster, Germany; 12 Department of Neurosurgery, University of Dresden, Dresden, Germany; 13 Department of Neurosurgery, University of Cologne, Cologne, Germany; 14 Department of Neurosurgery, University of Düsseldorf, Düsseldorf, Germany; 15 Department of Neurosurgery, Ludwig Maximillian University of Munich and German Cancer Consortium, Partner Site Munich, Munich, Germany; 16 Department of Neurosurgery, University Hospital RWTH Aachen, Aachen, Germany; 17 Department of Radiotherapy and Radiation Oncology, University of Leipzig, Leipzig, Germany; 18 Department of Radiation Oncology, University Hospital Bonn, Bonn, Germany; 19 Department of Neuroradiology, University Hospital Bonn, Bonn, Germany; 20 Department of Neuropathology, University Hospital Bonn, Bonn, Germany; 21 Clinical Study Core Unit Bonn, Institute of Clinical Chemistry and Clinical Pharmacology, University Bonn, Bonn, Germany; 22 Department of Neurology, University Hospital Heidelberg, Neurooncology Program at the National Center for Tumor Disease, German Cancer Consortium (DKTK), Clinical Cooperation Unit Neurooncology, German Cancer Research Center (DKFZ), Heidelberg, Germany

**Keywords:** Glioblastoma, Relapse, Meclofenamate, Temozolomide, Second-line therapy

## Abstract

**Background:**

Glioblastoma is the most frequent and malignant primary brain tumor. Even in the subgroup with O-6-methylguanine-DNA methyltransferase (MGMT) promoter methylation and favorable response to first-line therapy, survival after relapse is short (12 months). Standard therapy for recurrent MGMT-methylated glioblastoma is not standardized and may consist of re-resection, re-irradiation, and chemotherapy with temozolomide (TMZ), lomustine (CCNU), or a combination thereof. Preclinical results show that meclofenamate (MFA), originally developed as a nonsteroidal anti-inflammatory drug (NSAID) and registered in the USA, sensitizes glioblastoma cells to temozolomide-induced toxicity via inhibition of gap junction-mediated intercellular cytosolic traffic and demolishment of tumor microtube (TM)-based network morphology.

**Methods:**

In this study, combined MFA/TMZ therapy will be administered (orally) in patients with first relapse of MGMT-methylated glioblastoma. A phase I component (6–12 patients, 2 dose levels of MFA + standard dose TMZ) evaluates safety and feasibility and determines the dose for the randomized phase II component (2 × 30 patients) with progression-free survival as the primary endpoint.

**Discussion:**

This study is set up to assess toxicity and first indications of efficacy of MFA repurposed in the setting of a very difficult-to-treat recurrent tumor. The trial is a logical next step after the identification of the role of resistance-providing TMs in glioblastoma, and results will be crucial for further trials targeting TMs. In case of favorable results, MFA may constitute the first clinically feasible TM-targeted drug and therefore might bridge the idea of a TM-targeted therapeutic approach from basic insights into clinical reality.

**Trial registration:**

EudraCT 2021-000708-39. Registered on 08 February 2021

## Administrative information

Note: the numbers in curly brackets in this protocol refer to SPIRIT checklist item numbers. The order of the items has been modified to group similar items (see http://www.equator-network.org/reporting-guidelines/spirit-2013-statement-defining-standard-protocol-items-for-clinical-trials/).

**Table:** 

Title {1}	Phase I/II trial of meclofenamate in progressive MGMT-methylated glioblastoma under temozolomide second-line therapy – the MecMeth/NOA-24 trial
Trial registration {2a and 2b}.	EudraCT No. 2021-000708-39, registration date: 08 February 2021.
Protocol version {3}	AUTHOR Version \* MERGEFORMAT Version 2.0, 02.07.2021
Funding {4}	MecMeth/NOA-24 is an investigator-initiated trial. The trial is funded by the German Federal Ministry of Education and Research (grant 01EN2008). The trial is an official trial of the Neurooncology Working Group (NOA) of the German Cancer Society.
Author details {5a}	Thomas Zeyen^1^, Anna-Laura Potthoff^2^, Robert Nemeth^3^, Dieter H. Heiland^4,5^, Michael C. Burger^6^, Joachim P. Steinbach^6^, Peter Hau^7^, Ghazaleh Tabatabai^8^, Martin Glas^9^, Uwe Schlegel^10^, Oliver Grauer^11^, Dietmar Krex^12^, Oliver Schnell^4,5^, Roland Goldbrunner^13^, Michael Sabel^14^, Niklas Thon^15^, Daniel Delev^16^, Hans Clusmann^16^, Clemens Seidel^17^, Erdem Güresir^2^, Matthias Schmid^3^, Patrick Schuss^2^, Frank A. Giordano^18^, Alexander Radbruch^19^, Albert Becker^20^, Johannes Weller^1^, Christina Schaub^1^, Hartmut Vatter^2^, Judith Schilling^21^, Frank Winkler^22^, Ulrich Herrlinger^1^, Matthias Schneider^2^ ^1^Division of Clinical Neurooncology, Department of Neurology and Center of Integrated Oncology, University Hospital Bonn, Bonn, Germany ^2^Department of Neurosurgery and Center of Integrated Oncology, University Hospital Bonn, Bonn, Germany ^3^Institute of Medical Biometry, Informatics and Epidemiology, University Hospital Bonn, Bonn, Germany ^4^Department of Neurosurgery, University of Freiburg, Freiburg, Germany ^5^Faculty of Medicine, University of Freiburg, Freiburg, Germany ^6^Dr. Senckenberg Institute of Neurooncology, Goethe-University Hospital, Frankfurt am Main, Germany ^7^Department of Neurology and Wilhelm Sander-NeuroOncology Unit, University Hospital Regensburg, Regensburg, Germany ^8^Interdisciplinary Division of Neurooncology, University of Tübingen, Tübingen, Germany ^9^Division of Clinical Neurooncology, Department of Neurology, University Hospital Essen, Essen, Germany ^10^Department of Neurology, University Hospital Knappschaftskrankenhaus, Ruhr-Universität Bochum, Bochum, Germany ^11^Department of Neurology, University of Münster, Münster, Germany ^12^Department of Neurosurgery, University of Dresden, Dresden, Germany ^13^Department of Neurosurgery, University of Cologne, Cologne, Germany ^14^Department of Neurosurgery, University of Düsseldorf, Düsseldorf, Germany ^15^Department of Neurosurgery, Ludwig Maximillian University of Munich and German Cancer Consortium, Partner Site Munich, Munich, Germany ^16^Department of Neurosurgery, University Hospital RWTH Aachen, Aachen, Germany ^17^Department of Radiotherapy and Radiation Oncology, University of Leipzig, Leipzig, Germany ^18^Department of Radiation Oncology, University Hospital Bonn, Bonn, Germany ^19^Department of Neuroradiology, University Hospital Bonn, Bonn, Germany ^20^Department of Neuropathology, University Hospital Bonn, Bonn, Germany ^21^Clinical Study Core Unit Bonn, Institute of Clinical Chemistry and Clinical Pharmacology, University Bonn ^22^Department of Neurology, University Hospital Heidelberg, Neurooncology Program at the National Center for Tumor Disease, German Cancer Consortium (DKTK), Clinical Cooperation Unit Neurooncology, German Cancer Research Center (DKFZ), Heidelberg, Germany
Name and contact information for the trial sponsor {5b}	Rheinische Friedrich-Wilhelms-University of Bonn, represented by the Faculty of Medicine of the University of Bonn, represented by the Dean of the Faculty of the Medical Faculty, Venusberg-Campus 1, D-53127 Bonn, Germany Sponsor Delegated Person and Coordinating Investigator: Prof. Dr. med. Ulrich Herrlinger, Head Division of Clinical Neurooncology, Department of Neurology and Center of Integrated Oncology University Hospital Bonn Venusberg-Campus 1, D-53127 Bonn Tel.: 0049 228 28731241 Fax: 0049 228 28719043 Email: Ulrich.Herrlinger@ukbonn.de Representative of Coordinating Investigator/Principal Investigator: Dr. med. Matthias Schneider, Department of Neurosurgery and Center of Integrated Oncology University Hospital Bonn Venusberg-Campus 1, D-53127 Bonn Tel.: 0049 228 28710162 Fax: 0049 228 287 11366 Email: Matthias.Schneider@ukbonn.de
Role of sponsor {5c}	MecMeth/NOA-24 is an investigator-initiated trial. The financial sponsor (German Federal Ministry of Education and Research) had no role in the study design and will not have any role in collection, management, analysis and interpretation of data, writing and submission of the study report for publication. The legal sponsor Rheinische Friedrich-Wilhelms-University of Bonn with his sponsor-delegated person Prof. Ulrich Herrlinger and the MecMeth/NOA-24 study group consisting of the authors of the current publication, however, have ultimate authority over all of these activities.

## Introduction

### Background and rationale {6a}

Glioblastoma is known as the most frequent (3000 new cases/year in Germany) and most malignant (median overall survival (mOS) 17 months) primary brain tumor in adults [1, 2]. The subgroup of patients with a methylation of the MGMT promotor (approximately 1/3 of patients) exhibit a better clinical responding to alkylating chemotherapy with temozolomide (TMZ) or lomustine (CCNU) and thus have a longer mOS (26–48 months) than the subgroup of patients with non-methylated MGMT promotor. Nevertheless, recurrence occurs after a median period of roughly 16 months [3]. Here, a re-exposure to alkylating therapy is commonly applied, but the outcome is dismal with a median progression-free survival (mPFS) time of about 3 months and a mOS of 10–12 months after relapse [4, 5]. Thus, there is an urgent medical need for improved therapies for recurrent glioblastoma.

Previous preclinical studies have shown that glioblastoma cells exhibit ultra-long and thin membrane protrusions—dubbed tumor microtubes (TMs)—that extend into the surrounding (healthy) brain and tumor tissue in order to interconnect tumor cells over long distances [6]. TMs also interact with neurons through AMPAergic synapses which integrate the tumor cells into neural circuits and foster the malignant growth of these tumors by synaptic activity [7, 8]. The intercellular contacts are established by the aid of connexin-43 (Cx43)-based gap junctions that ultimately enable glioblastoma cells to arrange a multicellular functional network. Functional TMs, in turn, are associated with resistance to cytotoxic treatment through the formation of a self-repairing syncytial network which can be efficiently disturbed by downregulation of Cx43. Once the ability to establish TM networks is compromised, both TMZ chemotherapy and radiotherapy become much more efficient in preclinical glioblastoma models [6, 9].

In line with this, several studies demonstrated that pharmacological inhibition of gap junctions sensitizes primary MGMT-methylated human glioblastoma cell populations to TMZ-mediated toxicity [10–13].

However, a direct transfer into the clinical application has not been feasible so far, since none of the previously discussed gap junction inhibitors is suitable and available for clinical application.

Meclofenamate (MFA)—a nonsteroidal anti-inflammatory drug (NSAID) with clinical use in the USA—has been shown to exert distinct gap junction-inhibitory effects in various physiological cell types [14–16]. In addition, the formation of tumor-promoting glioblastoma-neuron synapses is inhibited by MFA [8]. Based on this knowledge, in our very recently published work [17], we aimed to investigate to what extent MFA might affect gap junction-mediated intercellular cytosolic traffic as well as electrical coupling in preformed glioblastoma networks. Our results show MFA to lead to both a morphological and a functional breakdown within glioblastoma network arrangements. This breakdown is driven by MFA-mediated inhibition of TM outgrowth and intercellular communication via two mechanisms: (1) a direct connexin-43 inhibition leading to significantly reduced intercellular gap junction-mediated cytosolic traffic and (2) a downregulation of adhesion and axon guidance molecules that spawns a reduction in the length of TMs leading to morphologically isolated tumor cells within the malignant network. This functional and morphological breakdown of glioblastoma intercellular networking resulted in a profound sensitivity to TMZ-mediated antitumor effects.

To summarize, preclinical results provide evidence for MFA to constitute the first potential TM-targeted drug that—with regard to its clinical approval as a NSAID in the USA—might be suitable for a clinical use. Based on this, the MecMeth/NOA-24 trial now explores the toxicity of a combination of MFA and standard TMZ, establishes a tolerable dose of MFA in this combination therapy in patients with relapsed MGMT-methylated glioblastoma (phase I), and ultimately searches for first signs of efficacy of this combination (phase II).

### Objectives {7}

In the phase I component, the primary objective is to determine the toxicity of MFA therapy in addition to standard TMZ and, on this base, to determine the daily MFA dose to be applied in a phase II component. Efficacy, safety, tolerability, and clinical effect on the quality of life of MFA in addition to standard TMZ throughout the trial constitute the secondary objectives.

In phase II, the main objective is to determine the efficacy of MFA therapy in addition to standard TMZ therapy. For this purpose, the dose determined in phase I will be used. Besides that, safety, tolerability, and clinical effect on the quality of life of MFA therapy in addition to standard TMZ will be investigated.

### Trial design {8}

The study consists of a single-arm phase I part and a randomized, parallel-group, and unblinded phase II part.

In phase I, patients with the first relapse of MGMT-methylated glioblastoma are screened for the trial. After inclusion, patients are pretreated with MFA in addition to standard TMZ and tumor resection will be performed 7–10 days after initiation of therapy. MFA tissue level measurements and translational analyses are performed on the tumor material obtained from resection. Each patient is particularly screened for dose-limiting toxicities (DLTs) during the first 8 weeks of his/her MFA treatment. Patients are recruited in two cohorts of three to six patients. Dose adaptation in between cohorts depends on the occurrence of DLTs as described below.

All patients of phase I are followed for toxicity and efficacy until the end of phase II.

In phase II, patients with the first relapse of MGMT-methylated glioblastoma are screened for the trial. After inclusion, patients are randomized (1:1) into two parallel groups, the experimental or the standard treatment arm. The experimental treatment is defined as treatment with MFA (dose determined in phase I) in addition to standard TMZ. The standard treatment arm is defined as monotherapy with TMZ (150–200 mg/m^2^ body surface). If tumor resection for the relapsed tumor is clinically indicated, the local treating neurosurgeon can decide whether the resection can be safely postponed until day 7–10 after initiation of therapy (optional, not a prerequisite for study participation in phase II) and tumor material for determination of MFA levels can be obtained.

MFA is taken at the dose determined in phase I. Daily MFA treatment is continued for 8 4-week courses (i.e., 224 days), until tumor progression, until the occurrence of a DLT attributable to MFA, or until definitive discontinuation of standard TMZ, whatever comes first. In case MFA therapy is terminated, standard TMZ therapy can be continued at the discretion of the treating physician.

## Methods: participants, interventions, and outcomes

### Study setting {9}

Phase I of the trial will be conducted in 10 centers in Germany (all academic hospitals), which must meet the structural and personnel requirements for performing the planned regular trial-related investigations. In phase II, the trial will be conducted in the 10 centers of phase I plus five additional German centers (all academic hospitals).

### Eligibility criteria {10}

Inclusion criteria for participants (phase I and II):

1. First relapse after first-line therapy with radiotherapy (RT) and alkylating chemotherapy, > 3 months after last chemotherapy application, and > 6 months after the end of RT. Drug therapy and/or radiotherapy for first relapse treatment not yet started
2. Tumor progression according to response assessment in neurooncology (RANO) criteria
3. Written informed consent
4. Cognitive state to understand rationale and necessity of study therapy and procedures
5. MGMT promotor-methylated, isocitrate-dehydrogenase (IDH) wildtype glioblastoma or gliosarcoma confirmed with histology of the primary resection
6. Age > 18 years
7. Karnofsky Performance Score (KPS) ≥ 60%
8. Life expectancy > 6 months
9. Adequate bone marrow reserve (white blood cell count (WBC) > 3 G/nl, platelets > 100 G/nl)
10. Adequate liver function (bilirubin < 1.5 × upper limit of normal (ULN); ASAT/ALAT < 3 × ULN, creatinine < 1.5 × ULN)
11. Patient compliance and geographic proximity that allow adequate follow-up
12. Male and female patients with reproductive potential must use an approved contraceptive method during and for 3 months after the trial (Pearl index < 1%)
13. Pre-menopausal female patients with childbearing potential: a negative serum pregnancy test (beta-HCG) must be obtained prior to treatment start

*Additional criterion for phase I:*

## Resection at first relapse not yet performed; according to the local treating neurosurgeon and the documented decision of local neurooncological tumor board, re-resection of the tumor is clinically indicated and can be safely deferred until day 7–10 after initiation of MFA/TMZ therapy

Exclusion criteria for participants (phase I and II):

1. Indication for hematotoxicity in first-line therapy not allowing TMZ starting dose 150 mg/m^2^/day
  Skin or liver toxicity > Common Terminology Criteria of Adverse Events (CTCAE) 5 grade 1 in first-line therapy
2. History of gastrointestinal (GI) bleeding or gastroduodenal ulcer, active gastritis
3. History of asthma, urticaria, or allergic-type skin reactions to NSAID
4. Prior malignancy other than glioma
5. History of confirmed or suspected hypersensitivity (delayed type and immediate type, inclusive of anaphylactic reaction) to any background/standard TMZ drug product or one of its ingredients of the chosen product, or to cyclooxygenase inhibitors (“NSAIDs”), or to any ingredient of meclofenamate drug product
6. History of disease with poor prognosis
7. Severe coronary heart disease (esp. after coronary artery bypass graft or history of myocardial infarction), severe heart failure
8. Known HIV infection, active hepatitis B or C
9. Breastfeeding or pregnant
10. Unable to undergo contrast-enhanced magnetic resonance imaging (MRI) (i.e., contrast allergy, implants, etc.).
11. Treatment in another clinical trial with therapeutic medical intervention or use of any other investigational agent during the trial or within the 30 days before enrolment
12. Medication with a drug that is not allowed in conjunction with MFA intake and cannot be discontinued, i.e., lithium, methotrexate, etc.
13. Patients with active bleeding, bleeding diathesis, antiplatelet therapy, or anticoagulant therapy except for the following anticoagulants which are permitted for low-dose thrombosis prophylaxis up to the dosage specified here: unfractionated heparin 7500 IU BID or 5000 IU TID; low molecular weight heparin, e.g., enoxaparin 40 mg/day; fondaparinux 2.5 mg/day; danaparoid sodium 750 IU BID; argatroban IV route thrombin time < 70 s; vitamin-K-antagonist INR < 1.8; dabigatran 150 mg BID; rivaroxaban 10 mg/day; edoxaban 30 mg/day; and epixaban 2.5 mg BID. This restriction is due to a potentially increased risk of GI ulcers with subsequent bleeding under MFA therapy.
14. Patients with medically diagnosed hereditary galactose intolerance, complete lactase deficiency, or confirmed glucose-galactose malabsortion
15. Medical history of gastrointestinal resection of any kind that may potentially alter the absorption of the investigational study drug, according to investigators’ judgment
16. The presence of any other concomitant severe, progressive, or uncontrolled renal, hepatic, hematological, endocrine, pulmonary, cardiac (including coronary artery bypass graft), or psychiatric disease, or signs and symptoms thereof, that may affect the subject’s participation in the study, according to investigators’ judgment

### Who will take informed consent? {26a}

Informed consent will be obtained by the investigator (a physician experienced for a minimum of 2 years in the care of brain tumor patients) of the respective center. The patient will receive an informed consent sheet where study rationale, design, risks, and potential benefits are precisely described. Additionally, there will be a detailed explanatory meeting with the investigator where this information will be explained and where the potential participant will have enough time to ask questions. Thereafter and after having read the informed consent sheet, the patient can give written informed consent. An adequate consideration time of 24 h will be given.

### Additional consent provisions for collection and use of participant data and biological specimens {26b}

Information on the collection and use of participant data and biological material (e.g., blood, tumor tissue) will be covered by a separate informed consent sheet.

## Interventions

### Explanation for the choice of comparators {6b}

In the treatment of relapsed MGMT promotor-methylated glioblastoma, standard chemotherapy may consist of TMZ or CCNU. Patients who relapse after a therapy-free time interval following the completion of first-line TMZ therapy may have a chance to respond again to TMZ therapy [5]; thus, TMZ therapy is justified as standard therapy in the standard arm.

### Intervention description {11a}

**Fig. 1:**
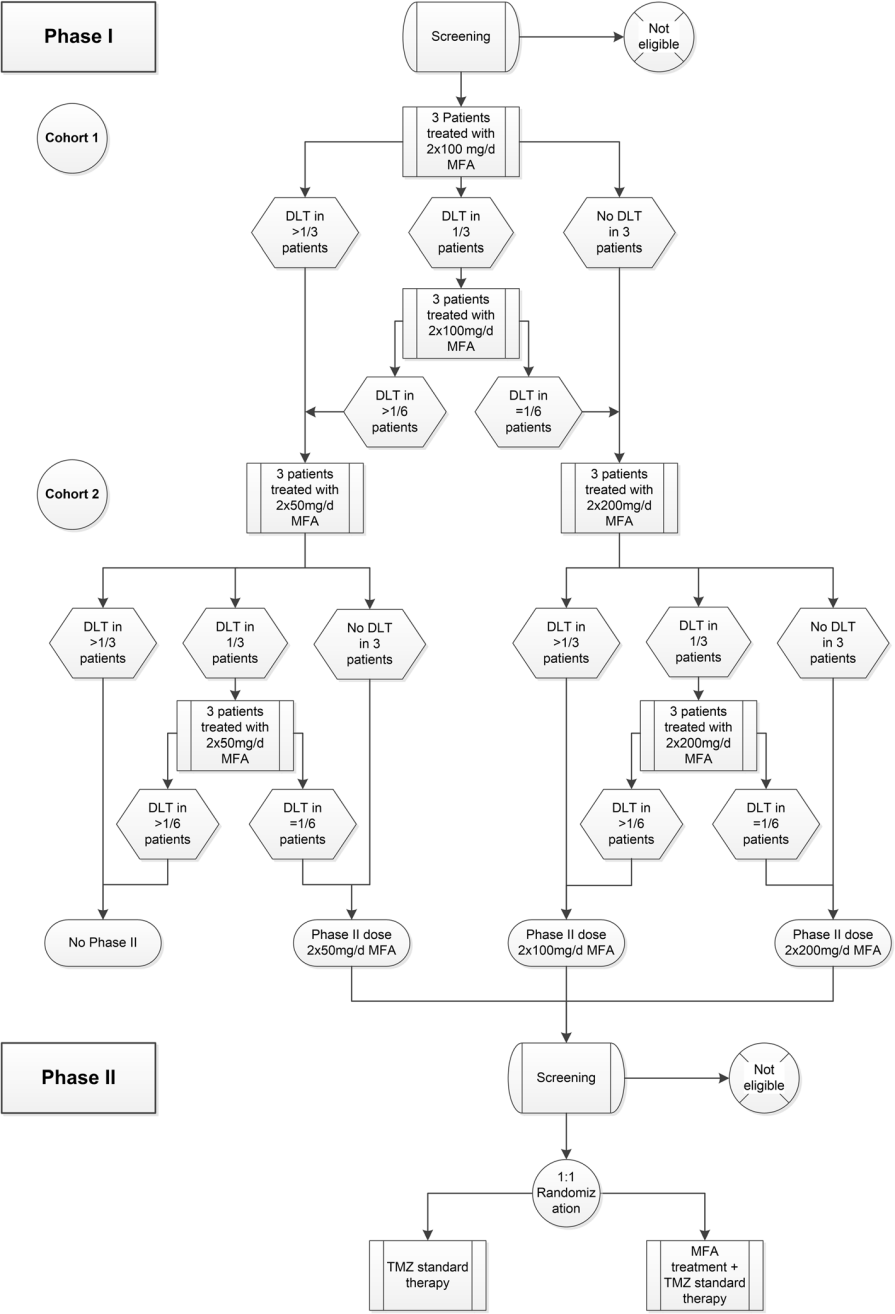
Dose-finding scheme in the MecMeth/NOA-24 trial

In phase I, there is one treatment arm and a dose-finding algorithm will be performed as described in the following. The participants will receive standard TMZ therapy for eight cycles (TMZ application orally on day 1–5 in a 28-day cycle, dose 150–200 mg/m^2^ body surface). In addition to that, the first three patients will receive 2 × 100 mg/day MFA daily (see also Fig. 1 dose-finding scheme). If no DLT occurs, the next three patients will be treated with 2 × 200 mg/day. Again, if no DLT occurs, this dose will be used for phase II. In both steps, if 1/3 patients develop a DLT, another three patients will be included. If there is a DLT in 1/6 patients, the dose will be elevated or the 2 × 200 mg/day dose will be used for phase II. If > 1 DLTs occur, the dose will be reduced in a next step to 2 × 50 mg/day and the same algorithm will be applied. If there are > 1 DLTs in 6 patients and the lowest dose of 2 × 50 mg/day, phase II will not be initialized. DLT is determined in the first 2 courses of MFA (within 8 weeks/56 (±3) days after first study treatment administration) in subjects with a compliance rate of 90% (i.e., 50 days of treatment) and no overdose. If judged to be related to the administration of MFA, the following toxicities will be considered a DLT: grade ≥ 4 hematotoxicity for > 14 days in courses 1 and 2; grade ≥ 3 for any other organ toxicity.

In phase II, the MFA dose determined in phase I will be applied. The potential participants will be screened. If they fit the inclusion criteria and give informed consent, there will be a 1:1 randomization. In the treatment arm, participants will receive standard TMZ + MFA (224 days at maximum). In the control arm, they will receive standard TMZ as described above.

### Criteria for discontinuing or modifying allocated interventions {11b}

For the individual patient, MFA therapy within the trial will be stopped if one of the following criteria applies:

1. Unequivocal MRI signs of disease progression according to RANO criteria
2. CTCAE5 grade 4 myelotoxicity/hematotoxicity for > 14 days in courses 1 and 2 related to MFA treatment
3. CTCAE5 grade 3+ for any other organ toxicity related to MFA treatment except for asymptomatic laboratory changes CTCAE5 grade 3
4. Definitive termination of standard TMZ therapy
5. Any event that leads to a delay in TMZ dosing lasting > 8 weeks from the beginning of the previous course of TMZ
6. Any adverse event, laboratory abnormality, or intercurrent illness which, in the judgment of the investigator, presents a substantial clinical risk to the subject with continued MFA application
7. Pregnancy
8. Lack of compliance of the subject (e.g., taking prohibited medication)
9. Significant protocol violations
10. Events probably related to the tumor resection/perioperative treatment or the underlying disease will not lead to discontinuation

### Strategies to improve adherence to interventions {11c}

To improve adherence to intervention protocols, the patient will be instructed to fill out a “patient’s diary” where MFA intake will be documented. This diary is also used to document co-medication, especially TMZ treatment and occurrence of adverse events (AEs). Apart from that, he or she will be requested to bring the empty containers/drug tablets of the MFA to the study visits.

### Relevant concomitant care permitted or prohibited during the trial {11d}

Besides standard TMZ therapy as described above, a prophylactic standard antiemetic regimen using a serotonin antagonist (e.g., ondansetron, tropisetron) prior to administration of standard TMZ is strongly advised. To keep the risk of gastrointestinal ulcers and/or bleeding as low as possible, all trial patients receiving MFA should be treated prophylactically with a proton pump inhibitor. Steroid medication as any other co-medication is performed at the discretion of the local investigator/treating physician. The administration of any other anticancer substance or other anticancer interventions is not permitted. However, it is prohibited to take drugs which can cause health risks when combined with MFA treatment, e.g., lithium or methotrexate. Considering the risk of potentially bleeding gastric ulcers under MFA, antiplatelet (acetylsalicylic acid, clopidogrel) and anticoagulant therapy is not permitted except for low-dose antithrombotic therapy.

### Provisions for post-trial care {30}

In case of progressive or recurrent disease, salvage therapy will be chosen at the discretion of the treating physician. Every subject participating in the trial is insured against any trial-related illness/injuries pursuant to the legal requirements which may occur during the trial. The investigator will inform the subject of the existence of the insurance, including the obligations arising from it.

### Outcomes {12}

In phase I, the primary outcome to be evaluated will be the incidence of DLTs during the first 8 weeks of MFA treatment. DLT for each dose level is defined as any CTCAE5 grade 3/4 organ toxicity or grade 4 hematotoxicity > 2 weeks during the first 8 weeks of MFA treatment; events probably related to the tumor resection and/or perioperative treatment or to the underlying disease are not regarded as DLT. Secondary outcomes in phase I are progression-free survival (PFS) as measured from the inclusion into the trial until diagnosis of progressive disease determined by MRI (according to “response assessment for neurooncology” (RANO) criteria) in the local center, PFS according to post hoc central reference neuroradiological assessment, overall survival (OS) as measured from the day of inclusion into the trial, assessment of safety beyond 8-week MFA treatment, and Karnofsky Performance Score (KPS), Quality of life (QoL), and Mini-Mental State Examination (MMSE) throughout the trial.

In phase II, the primary outcome to be evaluated will be PFS (randomization until the diagnosis of progressive disease according to RANO criteria). Secondary outcomes will be OS (randomization until death), continuous monitoring of AE/severe adverse events (SAE)/suspected unexpected serious adverse reactions (SUSARs), and quality of life throughout the trial (measured by KPS, QoLs, and MMSE). Survival analyses also include patients from phase I who received MFA at the same dose as applied in phase II.

### Participant timeline {13}

**Fig. 2:**
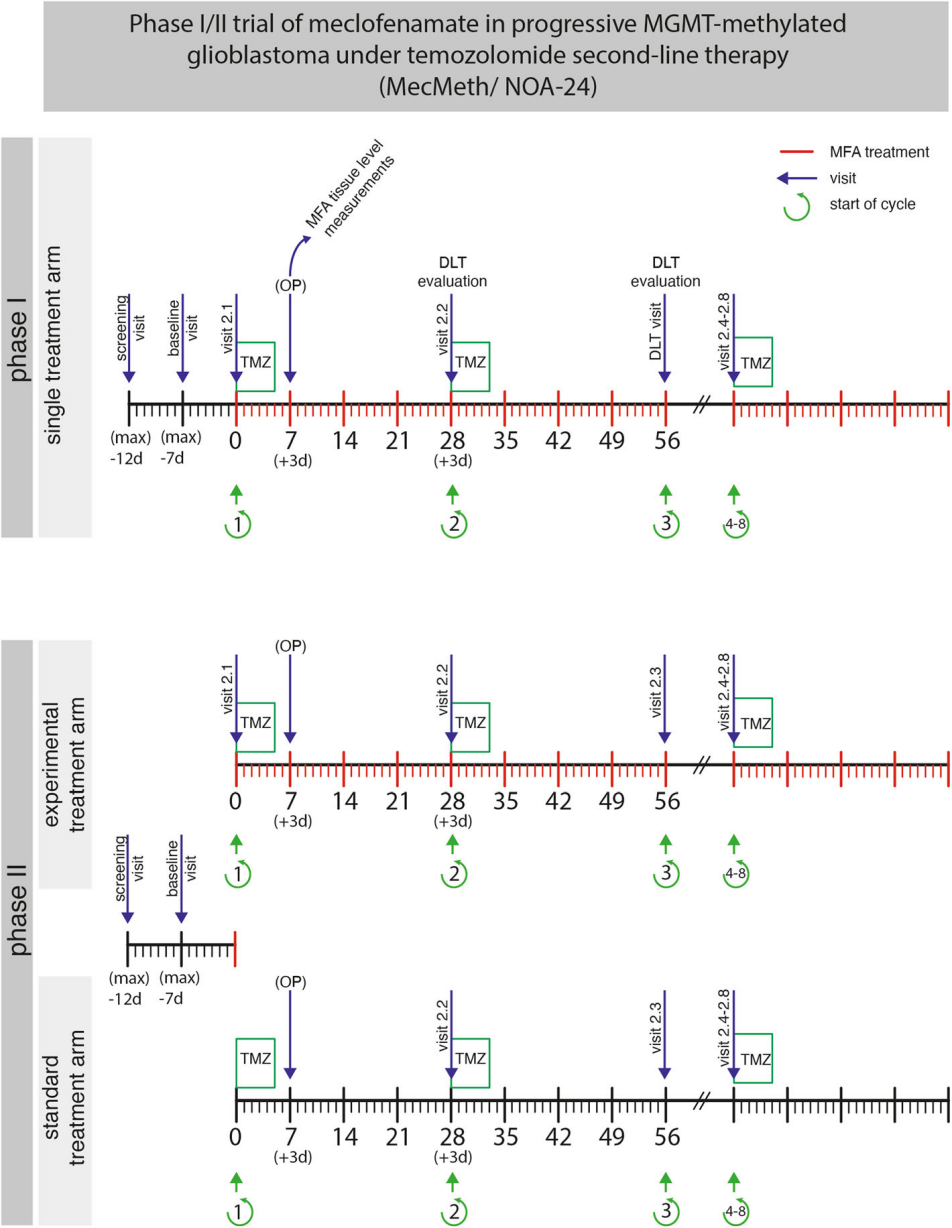
Timeline of visit and assessments

The participant timeline is illustrated in Fig. 2 and the extent of the visits is summarized in Table 1.

**Table 1:**
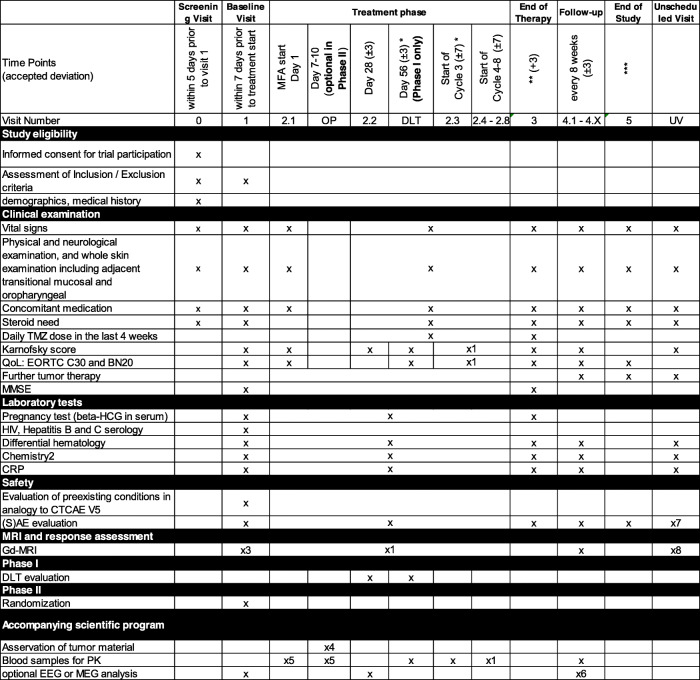
Visits and assessments in the MecMeth/NOA-24 trial

*DLT visit will only be performed in phase I. In phase I, visit 2.3 is only performed additionally; if the start of cycle 3 is prolonged for more than 3 days after the DLT visit, all patients in phase II have visit 2.3

**EoT: end of treatment is reached 3 days after last MFA intake (phase I and experimental arm of phase II) or on day 31 of the last TMZ cycle (standard arm of phase II)

***End of study and of follow-up in the entire trial will be reached when *both* of the following requirements are fulfilled: (1) at least 6 months after randomization of the last patient in phase II *and* (2) at least 3 days after definite termination of MFA intake in the last patient receiving MFA therapy (i.e., all patients have concluded study-related MFA intake for more than 3 days)

^1^Every 8 weeks: progression assessment, Karnofsky score and QoL, blood samples

^2^Na, K, creatinine, ASAT, ALAT, bilirubin

^3^Gadolinium-enhanced MRI obtained prior to inclusion/randomization can be used as baseline MRI if the interval between this MRI and the start of study therapy is shorter than 21 days. In patients who have undergone re-resection prior to randomization (only possible in phase II), the MRI used as baseline MRI has to be a postoperative MRI

^4^Relapse tumor resection 7–10 days after initiation of study therapy, tissue asservation should take place 2–4 h after the last intake of MFA/TMZ: (1) fresh frozen, (2) 4% PFA, and (3) FFPE tumor material for MFA/MFA metabolite level determination and for analysis of MFA-dependent tissue effects. The exact timepoint of the last MFA intake prior to resection and the exact timepoint of asservation of the tumor material have to be documented

^5^Timepoints (to be documented): (a) 2 h after first intake, (b) on the days between MFA start and resection daily blood sampling 2 h after morning application of MFA (optional), and (c) on the day of resection 5 blood samples at an interval of 2 h (0 h (prior to last preOP MFA dose) 2 h, 4 h, 6 h, 8 h later)

^6^Only at the first follow-up visit after discontinuation of MFA

^7^Only during ongoing MFA treatment + 3 days

^8^Only if clinically indicated

Briefly, patients start therapy within 7 days after inclusion/baseline visit. If according to the local treating neurosurgeon and the local neurooncological tumor board, re-resection of the tumor is clinically indicated and can be safely deferred until day 7–10 after initiation of MFA/TMZ therapy, tumor resection is performed 7–10 days after initiation of therapy. This is mandatory in phase I and optional in phase II. Resected tumor tissue is used for translational analyses of intratumoral MFA/MFA metabolite levels and detection of cellular and molecular effects of MFA. Also, blood analyses are performed for the determination of MFA blood levels 2 h after the first intake of MFA on day 1 and at 5 timepoints on the day of resection. During therapy, further visits are scheduled every 4 weeks prior to the start of a new TMZ cycle. These visits include clinical and standard laboratory examinations, documentation of co-medication documentation of (S)AE, KPS, and, every 8 weeks, contrast-enhanced MRI and QoL evaluation. In phase I, DLT evaluation is added at the visits prior to cycle 2 (day 28 ± 3) and cycle 3 (day 56 ± 3). MFA/TMZ treatment ends after 8 4-week cycles, at progression, or if MFA or at the timepoint when MFA or TMZ treatment has to be prematurely discontinued for whatever reason, e.g., intolerable toxicity, whatever comes first. After the end of the study therapy, patients are followed with clinical examination, contrast-enhanced MRI, and documentation of KPS, QoL, and further tumor therapy every 8 weeks. End of study and of follow-up in the entire trial will be reached when both of the following requirements are fulfilled: (1) at least 6 months after randomization of the last patient in phase II and (2) at least 3 days after definite termination of MFA intake in the last patient receiving MFA therapy (i.e., all patients have concluded study-related MFA intake for more than 3 days).

### Sample size {14}

Phase I uses an adaptive design based on DLTs (see Fig. 1). Starting with 3(-6) patients per dose level is standard for dose finding in phase I. Starting with a middle-level MFA dose of 2 × 100 mg/day (according to the US registration of MFA) ensures that only two dose levels have to be explored (*n* = 6–12 patients). Patients not evaluable for DLT or MFA tumor levels will be replaced (conservative estimate, 1/6 patients; thus maximal patient number in phase I, 12 + 2 = 14).

The randomized phase II part accrues 30 patients per arm. For the evaluation of PFS, the experimental arm also includes the 3–6 patients of phase I which have been treated with the same dose of MFA as in phase II for an additional analysis. Since phase II is searching for indications of efficacy but is not a confirmatory trial, dropouts will not be replaced. In glioblastoma trials, the dropout rate is relatively low (e.g., 9% in CeTeG/NOA-09 trial). Therefore, and since data from dropout patients are included as censored observation, the results may be expected to be robust regarding patients with short follow-up/early dropouts.

With 30 subjects per arm and a two-sided alpha level of 10%, we could detect a hazard ratio of approximately 0.44 if we assume a PFS-6 of 40% for the reference treatment and use a power of 80%.

### Recruitment {15}

Adequate participant enrolment will be achieved by continuous screening for potential participants in all participating centers. This is achieved by discussing every patient with a relapsed glioblastoma who is seen at a participating site in the interdisciplinary neurooncological tumor board which is mandated by German certification rules. This allows that all potentially eligible patients in a center can be identified.

## Assignment of interventions: allocation

### Sequence generation {16a}

The allocation to the treatment group in the phase II part will be performed electronically via the validated randomization tool of the electronic case report form (CRF) systemMARVIN (XClinical). The randomization will be performed using permuted blocks with a variable block size.

### Concealment mechanism {16b}

The usage of the electronic randomization tool in the electronic case report form (CRF) system described above ensures that the sequence is concealed until the intervention is assigned.

### Implementation {16c}

The allocation sequence is generated randomly by the computer-based MARVIN randomization tool implemented by the statistician of the Clinical Study Core Unit Bonn (SZB). After input of all data necessary for randomization (inclusion/exclusion criteria, demographic data), the electronic system displays the randomization result.

## Assignment of interventions: blinding

### Who will be blinded {17a}

In phases I and II, neither trial participants nor care providers will be blinded. However, neuroradiologists performing a post hoc re-analysis of the MRIs will be blinded for treatment protocol.

### Procedure for unblinding if needed {17b}

Unblinding will not be needed.

## Data collection and management

### Plans for assessment and collection of outcomes {18a}

Assessment and collection of baseline, outcome, and other trial data will be performed by the local investigator in the study visits and by evaluation of MRI scans by neuroradiologists. Every delegated assessor will be trained by a qualified trainer. The following study instruments will be utilized: questionnaires, laboratory tests, MRI scans, MMSE, (optional) electro-encephalography (EEG), or magnetic encephalography (MEG). A summary of the data to be collected is given in Table 1. Case report forms (CRFs) will be provided at the homepage of the clinical neurooncology division of the University of Bonn.

### Plans to promote participant retention and complete follow-up {18b}

To promote participant retention and complete follow-up, patients will be closely monitored. Besides, they will always have the chance to contact a responsible person at the site. Contact data (telephone numbers, email) will be given to the patient at the beginning of the trial. If participants decide to discontinue MFA treatment, they will be asked to stay in the follow-up for their own safety and to obtain follow-up data (AE, PFS, OS). If they are not willing to do so, the investigator is urged to ask the subject to return for an early termination visit and to document subject outcome (AE, PFS, OS), if possible. Any clinically significant abnormalities persisting at withdrawal will be followed by the investigator until resolution or until reaching a clinically stable endpoint. If a subject does not return for a scheduled visit, every effort will be made to contact the subject.

Subjects may also be withdrawn at any time at the discretion of the investigator for safety, behavioral, or administrative reasons. All patients who are discontinued from study therapy for any reason have to be followed according to the study protocol with clinical and MRI investigations until the end of the follow-up time of the whole trial (including the collection of all outcome data as described above).

### Data management {19}

Data management of the study will be carried out by the SZB (section Institute of Medical Biometry, Informatics and Epidemiology (IMBIE)). The study data is recorded and stored in a suitable, validated Clinical Data Management System (CDMS). Details on data management (procedures, responsibilities, data corrections, if any, which may be made by Data Management staff themselves, etc.) will be described in a *data management plan* prior to the trial. During the trial, the performance of data management and any deviations from the data management plan will be documented in a data management report. Queries and edit checks will be specified in a *data validation plan*. Before any data entry is performed, the trial database will be validated and the technical specifications of the database will be documented in a specific item list.

### Confidentiality {27}

The collection, transmission, archiving, and evaluation of personal data in this clinical trial are performed according to local applicable laws (Data Protection Act, General Data Protection Regulation). Prior to trial participation, each subject must be informed by the investigator about the purpose and extent of the collection and use of personal data, particularly medical data, and must give written informed consent.

The subjects must be informed that any subject-related data in this trial are handled confidentially and will be captured in pseudonymized form (subject ID number for the trial). It will only be transmitted to the coordinating investigator/sponsor/sponsor-delegated person/data monitoring safety board for scientific and adverse event evaluation and the responsible regulatory authority(ies) (local authority(ies)/BfArM or PEI), the Ethics Committees (ECs) of the trial sites and the European Data Base (EudraCT database) for verifying the proper conduct of the trial and for assessment of trial results and AEs.

During monitoring, audits, or inspections, representatives of the sponsor (monitor, auditor) or of the local regulatory authority(ies) must have direct access to personal data. In this case, the investigator is released from confidentiality. Consent to the collection and processing of personal data within the scope of this clinical trial can be withdrawn at any time. A patient is informed that she/he can terminate his/her participation in the clinical trial at any time—without giving reasons and without any following disadvantages. In the event of revocation of the declaration of consent, the data stored up to this point in time will continue to be used. This is necessary to determine the effects of the medicinal product under investigation and to ensure that the interests of the person concerned which are worthy of protection are not impaired, or to comply with the obligation to submit complete approval documents.

Individual participant data (including data dictionaries) that underlie results concerning primary or secondary endpoints reported in a published scientific article will be shared on demand after deidentification under the following conditions.

The data will be shared beginning 6 months and ending 3 years following article publication. Data are made available to researchers after a methodologically sound scientific proposal has been submitted to the coordinating investigator; a steering committee consisting of the coordinating investigator, the representative of the coordinating investigator, and a SZB member has approved the proposal; and a data access agreement has been signed. The study protocol and the informed consent forms will be made available on demand or through the website of the Division of Clinical Neurooncology, University of Bonn. After 36 months, the data will be available in our university’s data warehouse but without investigator support other than deposited metadata.

### Plans for collection, laboratory evaluation, and storage of biological specimens for genetic or molecular analysis in this trial/future use {33}

In phase I, patients receiving MFA and relapse tumor resection 7–10 days after initiation of study therapy will provide tumor material for determination of MFA/MFA metabolite tumor levels and analysis of cellular and molecular effects of MFA therapy according to internal standard operation procedures (SOPs) for tumor tissue acquisition and processing. MFA and metabolite measurements are performed under good clinical practice (GCP) conditions.

Patients receiving MFA and relapse tumor resection 7–10 days after initiation of study therapy will provide serum for determination of MFA/MFA metabolite serum levels on day 1 (2 h after first MFA intake) and on the day of tumor resection (prior to last MFA intake in the morning and 2 h, 4 h, 6 h, and 8 h later). Also, blood samples are drawn at baseline and at each timepoint with an MRI control to search for factors associated with tumor response, progression, and pseudoprogression.

## Statistical methods

### Statistical methods for primary and secondary outcomes {20a}

In phase I, the primary endpoint toxicity will be defined as incidence of DLTs and subsequent determination of the optimal dose for phase II and does not require extensive statistical analysis. To have robust results and the required number of patients with all necessary data for determining the phase II dose, early dropout patients/patients with insufficient data for 8-week DLT will be replaced. For secondary endpoints, additional analyses will include a post hoc analysis of the primary endpoint PFS as reviewed centrally by a neuroradiologist (Department of Neuroradiology, University Hospital Bonn).

In phase II, the primary outcome PFS is analyzed using a two-sided log rank test and a Cox regression analysis to estimate the hazard ratios in the intention-to-treat (ITT) population. PFS is measured from the day of randomization until the diagnosis of progressive disease determined by MRI (RANO criteria) in the local center.

OS is analyzed using a two-sided log rank test and a Cox regression analysis to estimate the hazard ratios in the ITT population. OS is measured from the day of randomization until death. Descriptive Cox regression analyses for common prognostic factors (age, KPS) and subgroup analyses for categorized common prognostic factors are also included. Phase II also includes descriptive statistics of all AEs (with reference to the number of patients and number of MFA/TMZ courses applied) during therapy. Dose reductions of TMZ, delay of TMZ therapy in subsequent courses, and premature withdrawals and development of KPS throughout the trial will also be described, as well as MFA/MFA metabolite tumor tissue and serum levels in patients resected after the start of MFA therapy are described.

QoL will be recorded using the standardized and validated EORTC QLQ-C30 and BN20 questionnaires [18]. The scores for the different dimensions will be compared between repeated assessments throughout the trial and between the two arms of the trial. As proposed in previous trials [19–21], domains of particular interest for brain tumor patients are preselected for particular consideration (global health status, physical functioning, social functioning, cognitive functioning, communication deficit, and motor dysfunction).

Additional details of the statistical analysis will be specified in the statistical analysis plan of the trial.

### Interim analyses {21b}

In phase I, an interim analysis is planned when the first and second cohorts have been treated for 56 days with MFA. The interim analysis and interim report will describe subject recruitment, treatment compliance, and safety, tolerability, and the occurrence of any DLT for the subjects. Efficacy parameters will not be analyzed. After data cleaning and analysis, the interim report will be submitted to the data and safety monitoring board (DSMB) to obtain its advice regarding the MFA dose recommended for phase II. Phase II does not include an interim analysis.

### Methods for additional analyses (e.g., subgroup analyses) {20b}

Additional sensitivity analysis will be performed for survival endpoints in phase II (PFS and OS) including also the patients of the phase I part who received the same dose of MFA.

### Methods in analysis to handle protocol non-adherence and any statistical methods to handle missing data {20c}

In phase I, patients who are not evaluable for DLT will be replaced. A patient is evaluable for DLT if the DMSB has determined that a DLT has occurred in this patient during the first 56 days after treatment start (independent of the duration of MFA intake) or if until DLT visit (day 56 ± 3 days after start of MFA treatment) the patient has taken at least 100 doses of MFA (i.e., 89.3% of the maximally positive cumulative dose in 56 days) as documented by the patient’s diary and the documented return of 1 empty container of MFA (containing 100 capsules) (for dose step 2 × 100 mg daily or 2 × 50 mg daily) or of 2 empty containers of MFA (for dose step 2 × 200 mg daily).

In phase II, subjects dropping out of the trial prior to randomization will be listed as screening failures including the reason for dropout. Subjects prematurely discontinuing the trial will be censored regarding the analysis of PFS or OS at the time of discontinuation. Subjects dropping out after randomization will be analyzed using all available data and will not be replaced. The technique for the analysis of missing values will be defined in the statistical analysis plan. A check of a possible treatment effect on the frequency of missing values will be performed.

### Plans to give access to the full protocol, participant-level data, and statistical code {31c}

The study protocol and the informed consent forms will be made available on demand or through the website of the Division of Clinical Neurooncology, University of Bonn. Apart from that, the statistical analysis plan and the clinical study report will be made available. Participant-level data will be shared beginning 6 months and ending 3 years following article publication. Data are made available to researchers after a methodologically sound scientific proposal has been submitted to the coordinating investigator; a steering committee consisting of the coordinating investigator, the representative of the coordinating investigator, and a SZB member has approved the proposal; and a data access agreement has been signed. After 36 months, the data will be available in our university’s data warehouse but without investigator support other than deposited metadata.

## Oversight and monitoring

### Composition of the coordinating center and trial steering committee {5d}

To ensure accurate, complete, consistent, and reliable data, the investigator’s site(s) and trial procedures will be monitored by a representative of the sponsor.

The sponsor’s representative or his delegate will visit the site and oversee the following things: evaluation of the progress and recruitment of the trial; review of source documents and case report forms (CRFs) for protocol compliance, accuracy, and validation; assessment of facilities and equipment; check for protocol compliance; check for correct and timely AE/SAE and DLT reporting; verifying of proper handling and dispensing of the investigational medicinal product (IMP); and other factors.

Source data verification will be performed in order to verify the accuracy and completeness of the entries on the CRF by comparing them with the source data, and to ensure and increase the quality of the data. All data which are subject to source data verification (SDV) must have been entered in the medical record or, in the case of source documents, enclosed with the medical record. The investigators will provide access to the medical records for the performance of SDV.

Frequency and scope of the monitoring visits will be defined in the Monitoring Plan for this trial which also includes the extent of source data verification that is required.

### Composition of the data monitoring committee, its role, and reporting structure {21a}

There will be an independent data and safety monitoring board consisting of a neurooncologist, a neurosurgeon, a medical oncologist, and a statistician. It will review safety data and recruitment data. It will also provide a recommendation for the further proceeding of the trial to the sponsor. Meetings of this board are planned for evaluating DLT data of phase I after each dose step when all patients in this dose step are evaluable for DLT, about 6 months after recruitment of the first patient of phase II, and after all patients have concluded MFA therapy. Of course, the sponsor or the DSMB itself can schedule additional meetings at any timepoint.

### Adverse event reporting and harms {22}

Any AE and SAE (per definition in the trial protocol) has to be documented in the CRF on the respective adverse event report form. AE/SAE documentation will be performed for each participant between the baseline visit and the visit at least 3 days after termination of MFA administration. AEs will be reported and evaluated according to the Common Terminology Criteria for Adverse Events (CTCAE5). According to the GCP-V, only AEs and unexpected clinical diagnostic findings that are identified in the protocol as critical for clinical trial evaluation must be documented and transmitted to the sponsor. To ensure comparability of the safety data of the two study arms, any adverse medical incident in patients of the control arm will also be recorded although no study drug will be administered. Whenever possible, diagnoses should be given when signs and symptoms are due to a common etiology. All measures required for adverse event management must be recorded in the source document and reported according to sponsor’s instructions. The investigator evaluates all AEs regarding severity, causality, and seriousness. Every SAE has to be reported immediately via fax to the Study Coordinating Center at the SZB. To take into account safety data available to the sponsor but not to the investigator at the time the SAE was detected, in addition to the initial assessment of a SAE by the investigator, a second assessment of the event by the sponsor in terms of causality and probability of occurrence (“expectedness”) and a continuous benefit-risk assessment are performed.

### Frequency and plans for auditing trial conduct {23}

This trial may be selected for audit by representatives of the sponsor or, independent from the sponsor, for inspection by site responsible representatives of the local regulatory authority. There is no predefined schedule for audits and inspections. After every audit, the auditee(s) will receive an audit confirmation by the auditor. This document has to be filed together with the trial documentation and has to be made available also to the authorities in case of an inspection. At the end of the trial, a copy of the audit certificate(s) will be included in the final report.

### Plans for communicating important protocol amendments to relevant parties (e.g., trial participants, ethical committees) {25}

The sponsor can implement changes to the protocol after the clinical trial has started. These may be administrative (logistical/administrative amendments) or substantial. Substantial amendments (e.g., those who can affect the safety or physical or mental integrity of participants or the scientific value of the trial) require a new authorization of the national competent authority and a new favorable opinion by the Ethics Committee (EC). The participants will be informed about substantial amendments and, if applicable, an updated Informed Consent Form has to be signed by all subjects enrolled in the trial who are affected by the amendment.

Amendments which only have to be approved by the EC (e.g., changes in an advertisement for subjects to participate in the trial or changes in facilities for the trial) also will be notified to the national competent authorities (NCA) with the comment “For information only.” Similarly, the EC will be informed of any substantial amendments for which only the NCA is responsible (e.g., quality data). If administrative protocol changes (e.g., change of monitoring, telephone numbers) are necessary, the EC and NCA will be notified only. All amendments will be communicated to the participating trial centers.

## Dissemination plans {31a}

Trial results will be made accessible via presentation at scientific meetings and written publication in a scientific journal and, as required by the EU Commission Guideline 2012/C 302/03, by reporting in the EudraCT database. After completion of the analysis by the responsible biostatistician, the final integrated medical and statistical report will be prepared and submitted to NCA and EC. According to EU Directive 2001/20/EC, a lay summary will be created and published in the EudraCT database. Except when required by law, no one will disclose a result of the clinical trial to third parties unless all relevant parties involved have first agreed on the results of the analysis and their interpretation. All data collected in connection with the clinical trial will be treated in confidence by the sponsor/coordinating investigator and all others involved in the trial, until publication. Interim data and final results may only be published (orally or in writing) with the agreement of the coordinating investigator as the sponsor-delegated person. Individual participant data will be available. Individual participant data (including data dictionaries) that underlie results concerning primary or secondary endpoints reported in a published scientific article will be shared on demand after deidentification (see the section “Confidentiality {27}”). Furthermore, the following documents will be made available: study protocol, statistical analysis plan, informed consent form, and clinical study report. The data will be shared beginning 6 months and ending 3 years following article publication. Data are made available to researchers after a methodologically sound scientific proposal has been submitted to the coordinating investigator; a steering committee consisting of the coordinating investigator, the representative of the coordinating investigator, and a member of the SZB has approved the proposal; and a data access agreement has been signed. The study protocol and the informed consent forms will be made available on demand or through the website of the Division of Clinical Neurooncology, University of Bonn. After 36 months, the data will be available in our university’s data warehouse but without investigator support other than deposited metadata.

## Discussion

All practical and operational issues have been described above.

## Trial status

The beginning of recruitment is planned for August/September 2021 and will be approximately completed by February 2024.

## Abbreviations

MGMT: O-6-Methylguanine-DNA methyltransferase
TMZ: Temozolomide
CCNU: Lomustine
MFA: Meclofenamate
NSAID: Nonsteroidal anti-inflammatory drug
mOs: Median overall survival
mPFs: Median progression-free survival
TM(s): Tumor microtubes
Cx43: Connexin 43
DLTs: Dose-limiting toxicities
CTCAE: Common Terminology Criteria of Adverse Events
RANO: Response assessment in neurooncology
SOP: Standard operation procedure
ITT: Intention to treat
IMP: Investigational medicinal product
SDV: Source data verification
EC: Ethics Committee
RT: Radiotherapy
NCA: National competent authorities
BfArM: Bundesinstitut für Arzneimittel und Medizinprodukte
AEs: Adverse events
KPS: Karnofsky Performance Score
MMSE: Mini-Mental State Examination
QoL: Quality of life
SAE: Serious adverse event
SUSARs: Suspected unexpected serious adverse reactions
CRF: Case report form
MRI: Magnetic resonance imaging
SZB: Studienzentrale Bonn = study core unit Bonn
IMBIE: Institute of Medical Biometry, Informatics and Epidemiology
IIT: Investigator-initiated subject
DSMB: Data and safety monitoring board
GCP-V: Good clinical practice V IDH Isocitrate-dehydrogenase WBC White blood cell count ULN Upper limit of normal BID “bis in die” = zweimal täglich
TID: “ter in die” = dreimal täglich
GI: Gastrointestinal
EEG: Electro-encephalography
MEG: Magnetic encephalography
CDMS: Clinical Data Management System
